# Smart match: revolutionizing organ allocation through artificial intelligence

**DOI:** 10.3389/frai.2024.1364149

**Published:** 2024-02-28

**Authors:** Rajkiran Deshpande

**Affiliations:** Department of Renal Transplant and General Surgery, Manchester Royal Infirmary, University Manchester Hospital NHS Foundation Trust, Manchester, United Kingdom

**Keywords:** organ procurement, transplantation, artificial intelligence, organ utilization, organ allocation

## Abstract

In this transformative era of organ transplantation, integrating Smart Match and artificial intelligence (AI) emerges as a pivotal advancement, revolutionizing organ allocation processes. Smart Match employs AI algorithms, enhancing organ matching precision and optimizing transplantation outcomes. Leveraging machine learning addresses complexities in donor-recipient pairing, immunosuppression management, and post-operative care, promising to minimize waitlist mortality and improve patient wellbeing. The multifaceted potential of Smart Match lies in its ability to not only streamline current practices but also pave the way for future innovations in solid organ transplantation. As technology continues to evolve, the collaboration between Smart Match and AI exemplifies a beacon of progress, promising increased efficiency, equitable organ distribution, and improved patient care. This article delves into the paradigm shift facilitated by Smart Match and AI, emphasizing their transformative impact on the landscape of organ allocation and patient outcomes.

## 1 Introduction

Organ transplantation has a rich history, starting with the first human kidney transplant in 1954. Subsequent decades saw milestones in liver, pancreas, heart, lung, and intestine transplants (Terry Sharrer, 2022). Early successes resulted from advances in research, methodologies, and surgical techniques, with immunological challenges being initial setbacks. Evolving with immunosuppressive medications and surgical innovations, organ transplantation is now standard care for end-stage organ diseases (Nordham and Ninokawa, 2022). Ongoing research, such as face transplants and lab-grown organs, pushes boundaries (Bezinover and Saner, 2019). Commitments to organ transplantation aim to extend life expectancy, enhance clinical conditions, and improve overall quality of life (Grinyo, 2013). The challenges in kidney organ allocation include increasing marginal deceased donors (MDD), rising cold ischemia times (CIT), inadequate planning and resources, and the need for flexible allocation and transportation systems. The surge in deceased donors poses challenges in kidney transplantation, causing extended cold ischemia times and increased discard rates. The allocation system's uniform treatment of MDD kidneys overlooks a recognized “hard-to-place” phenotype. A flexible allocation system is crucial for optimizing organ utilization. Early identification and a “fast-track” policy for MDD kidneys can significantly enhance utilization rates. Balancing efficiency and fair access involves factors like Human Leucocyte Antigen (HLA) mismatch, CIT, dialysis duration, donor-recipient age, and comorbidity (Wu et al., 2017; Stratta, 2022). The evolving allocation landscape prioritizes factors beyond HLA mismatch, enhancing equity and transparency in transplantation (Ross et al., 2012). Innovative strategies, like survival prediction scores and matching based on predicted graft and recipient survival, contribute to optimal outcomes and equitable distribution. Patient preferences, favoring younger recipients and well-matched donors, shape ongoing discussions on kidney allocation. This multifaceted approach underscores the complexity and continuous evolution of organ allocation strategies for optimal outcomes and equitable distribution (Stratta, 2022). Artificial Intelligence (AI), powered by classifiers like neural networks, decision trees, random forests, and naive Bayes, transforms organ allocation by predicting outcomes and optimizing donor selection (Ivanics et al., 2020). With demonstrated superiority in immunosuppression management and graft survival assessment, integrating AI into electronic medical records is crucial for widespread adoption, paving the way for its pivotal role in revolutionizing organ allocation (Schwantes and Axelrod, 2021).

## 2 Methodology

In this minireview, the author acknowledges that an exhaustive review of all databases and articles on AI in organ transplantation was not undertaken due to its focused nature. It systematically gathers information on integrating artificial intelligence (AI) into organ transplantation. Keyword searches, including “AI in organ transplantation,” “organ allocation,” and “transplant outcomes,” were conducted across reputable databases like PubMed and ScienceDirect. Additionally, data from Eurotransplant and the United Network for Organ Sharing (UNOS) were included to ensure a comprehensive overview. A content analysis of peer-reviewed articles from 2013 to 2023 was done. This synthesis formed the foundation for the article, presenting an overview of historical contexts, transplantation challenges, and AI's role in addressing these issues.

## 3 Artificial intelligence and optimizing organ utilization

Artificial intelligence (AI) and machine learning (ML) rest on fundamental principles, aiming to create intelligent machines capable of learning and adapting. AI seeks to build smart systems, while ML, a subset of AI, involves training models to learn and improve over time. Core principles encompass learning, reasoning, problem-solving, perception, and language comprehension. ML utilizes methods like supervised (learning from labeled data), unsupervised (finding patterns without predefined labels), and reinforcement learning (agents making decisions through trial and error) (Delgado De Molina Rius, 2023). These principles form the basis of AI and ML, offering powerful problem-solving tools across different fields.

### 3.1 State of the art algorithms

Artificial Intelligence (AI) revolutionizes organ transplantation through machine learning for improved donor-recipient matching. The process involves aligning characteristics for successful outcomes. Eurotransplant and the United Network for Organ Sharing (UNOS) utilize allocation systems based on expected outcomes and emergencies, considering the limited viability timeframe of harvested organs. Existing systems like Child-Pugh, Model of End Stage Liver Disease (MELD), Kidney Allocation System (KAS), and Lung Allocation System (LAS) lack real-time prioritization and require continuous modifications. AI integration ensures optimal decision-making, maintaining equal access amid organ scarcity and growing waiting lists (Peloso et al., 2022).

Bertsimas et al. (2019) proposed OPOM (Optimal Prediction of Mortality), a machine learning model derived from the OCT algorithm for liver allocation, outperforming MELD in predicting 3-month mortality or waitlist removal. It included parameters such as sex, race, cause of liver failure, demographics, cumulative wait time, blood type, average age, and BMI. The outcome was a classification tree predicting the probability of a patient's death or unsuitability for transplant within 3 months (dependent variable) based on specific patient characteristics (independent variables). With a dataset of 1,618,966 observations, OPOM reduced mortality by 417.96 deaths in 6,139 liver transplantations. External validation is pending.

Cruz-Ramírez et al. (2013) conducted a study that aims to optimize organ allocation in liver transplantation using a machine-learning approach. The memetic Pareto evolutionary non-dominated sorting genetic algorithm 2 (MPENSGA2) was employed to train radial basis function neural networks, considering donor, recipient, and organ characteristics to predict graft survival for 3 months after the transplant. Results showed competitive performance across various metrics, with the multi-objective algorithm outperforming the mono-objective one. The rule-based system, developed from neural network models, complemented the allocation system (MELD) in 55% of cases, emphasizing efficiency and equity.

A Large Spanish multicentre study [Model for Allocation of Donor and Recipient in España (MADR-E)] by Briceño et al. (2014) proposed using artificial neural networks (ANNs) for donor-recipient (D-R) matching in liver transplantation to address the shortage of available organs. Analyzing 1,003 liver transplants from 11 Spanish centers, the study employs 64 donor and recipient variables, developing two ANN models, NN-CCR (Neural Network for Correct Classification Rate) and NN-MS (Neural Network for Minimum Sensitivity), using the Neural Net Evolutionary Programming algorithm. Results show superior performance in predicting graft survival (90.79%) with AUC 0.81 and graft loss (71.42%) with AUC 0.82 compared to traditional models and established scores. Receiver-operating curves highlight the heightened accuracy of ANNs in predicting 3-month graft outcomes. The study suggests that ANNs serve as a powerful decision-making tool, optimizing principles of justice, efficiency, and equity, with potential applications in predicting short-term outcomes and as a research avenue for future D-R matching models.

Rana et al. (2008) developed Survival Outcome Following Liver Transplant (SOFT) score, integrating recipient and donor characteristics based on Organ Procurement and Transplantation Network (OPTN)/Scientific Registry of Transplant Recipients (SRTR) data. Unlike MELD, SOFT accurately predicts 3-month recipient survival after liver transplantation. The analysis identified 18 risk factors, excluding warm ischemia, with key predictors being previous transplantation and pretransplant life support. Combined with MELD, SOFT enables real-time decision-making for transplant candidates, offering improved quantification of survival benefits for individual procedures. The accuracy of SOFT score in predicting outcomes is comparable to other models, as indicated by its C-statistic of 0.70 (Jacob et al., 2005; Ioannou, 2006).

Yasodhara et al. (2021) utilized machine learning algorithms like GBS (Gradient Boosting Survival) to assess liver transplant outcomes. Their study identified diabetes mellitus (DM), not obesity, as a superior predictor for transplant outcomes. Analyzing data from the Scientific Registry of Transplant Recipients (SRTR) and a University Health Network (UHN) cohort of 18,058 liver transplant recipients, the study revealed increased serum creatinine and hypertension significantly impacted mortality in patients with pre-existing DM. Despite limitations like retrospective design, incomplete comorbidity data, unclear immunosuppression information, and a low number of patients with steatohepatitis, this study, among the largest in its field, emphasizes diabetes as a more reliable indicator than obesity in donor-recipient matching outcomes, contributing valuable insights into liver transplantation risk factors.

Bae et al. (2019) introduced an online tool (https://www.transplantmodels.com/kdpi-epts/) utilizing AI-ANNs algorithms for optimizing kidney donor-recipient matching. The tool estimates 5-year patient survival by employing a random survival forest (RSF) and combining the expected post-transplant survival (EPTS) score and Kidney Donor Profile Index (KDPI). The RSF algorithm achieved a C-statistic of 0.637, slightly surpassing the Kidney Donor Risk Index (KDRI) model's 0.6. This predictive model can enhance personalized decision-making for kidney offers in clinical practice.

Artificial intelligence (AI) automates evaluations, ensuring faster and more accurate assessments (Alamgir et al., 2022). It aids in predicting graft survival based on diverse parameters like donor's eGFR, recipient and donor BMI, recipient-donor weight difference, and additional factors like donor's age, gender, kidney donor profile index (KDPI), estimated post-transplant survival (EPTS), and kidney donor risk profile (KDRI). AI accurately predicts delayed-graft function (DGF), offering valuable risk assessment insights (Konieczny et al., 2021). It identifies patterns, optimizes donor-recipient matching, and predicts waiting list mortality and graft survival. Integrating AI into electronic medical records and organ offer systems is crucial for adoption. AI models outperform traditional metrics like the Model for End-Stage Liver Disease (MELD) score in donor allocation success rates. However, full AI integration in liver transplantation needs validation through multicentre randomized controlled trials to ensure efficacy and address clinician resistance (Bhat et al., 2023). AI systems use preference modeling and social choice techniques aligned with human values. These systems learn and aggregate people's preferences to guide AI behavior, ensuring that the learned preferences are accurate. However, people are often indecisive, especially when decisions have moral implications (McElfresh et al., 2021). To address indecision, mathematical models based on philosophy, psychology, and economics have been formalized to describe agent decisions (Peloso et al., 2022).

### 3.2 AI addressing unmet needs

In 2021, kidney transplant recipients constituted the largest group, with over 24,000 individuals receiving kidney transplants out of a total of 41,354 organ transplants. Despite this, a substantial challenge persists as approximately 66,000 eligible kidney transplant patients remained on the extensive 106,000-patient waitlist, and 24.5% of donated kidneys (6,427) were not transplanted, presenting a considerable challenge in meeting this demand (Dageforde et al., 2023). The discard rate increases exponentially with organ quality, notably higher Kidney Donor Profile Index (KDPI) scores (Alhamad et al., 2019). This emphasizes a significant opportunity for improved kidney utilization, particularly from older donors with more comorbidity (Aubert et al., 2019). Transplantation has been demonstrated to be a cost-effective and often cost-saving procedure for suitable candidates, even with lower-quality organs, which can improve quality and quality of life. AI decision support enhances kidney utilization by aiding clinician decision-making and providing real-time access to data-driven predictions, promising to alleviate the unmet need for kidney transplants, especially for higher Kidney Donor Profile Index (KDPI) scores (Axelrod et al., 2018). AI optimizes donor-recipient matching in transplantation, ensuring more precise matches beyond traditional metrics. Predictive algorithms enhance accuracy in forecasting post-transplant outcomes, allowing dynamic organ allocation based on individual patient profiles. AI also supports risk assessment, predicting factors like delayed graft function, contributing to improved patient outcomes. Ultimately, AI has the potential to enhance efficiency and equity in organ allocation, maintaining principles of justice, efficiency, and equity in transplantation (Gotlieb et al., 2022).

### 3.3 Smart match system

The Smart Match system in AI for organ transplantation integrates various components and architecture to revolutionize the transplantation process. A key element is the Intelligent Match Making Assistant (IMMA), employing Case-Based Reasoning (CBR) techniques, including Case Retrieval Nets and the Spreading Activation Algorithm. IMMA's role is crucial in searching the patient waiting list and investigating Donor-Recipient Compatibility, ultimately enhancing the efficiency of the Human Organ Transplantation Management (HOTM) system (Schwantes and Axelrod, 2021). The Internet of Things (IoT) is another pivotal component. In the organ procurement system, wireless sensors monitor organ status, transmitting real-time data to cloud-based servers via microcontrollers and healthcare-specific gateways. For instance, a sensor on a donated kidney tracks its temperature, ensuring preservation. This IoT-driven system optimizes organ procurement, offering accurate information and addressing ethical, legal, and clinical considerations. Integrated with the National Organ Donation/Transplantation Registry System (NODTRS), it adopts information technology and security methodologies, ensuring the secure and dependable functioning of the registry. Collectively, these components contribute to improving patient outcomes and increasing the utilization of healthy organs for transplantation (Schwantes and Axelrod, 2021).

## 4 Discussion

Integrating artificial intelligence (AI) in organ allocation presents numerous benefits, notably enhancing accuracy in donor-recipient matching, reducing waiting times, and elevating overall transplant success rates. AI's analytical prowess enables more precise compatibility assessments, streamlining the allocation process. Furthermore, its potential extends to mitigating disparities in organ access related to geographic location or socioeconomic factors.

Eurotransplant and the United Network for Organ Sharing (UNOS) utilize allocation systems based on expected outcomes and emergencies. When a transplant hospital identifies a candidate, it inputs medical data, including blood type and urgency, into the UNOS system. Simultaneously, organ procurement organizations provide donor data. Based on this information, UNOS generates a unique rank-ordered list, prioritizing candidates in urgent need or with higher chances of survival (US Organ Donation System, 2024). Eurotransplant's centralized database enables member state transplant centers to enter patient and donor information, initiating an international waiting list. When a donor becomes available, a complex algorithm factors in medical and ethical criteria to generate a match list, facilitating efficient organ allocation within the Eurotransplant network (Eurotransplant, 2024). Although these systems are integral to clinical practice, they do not provide real-time prioritization, and their effectiveness requires ongoing modifications. The disparity between transplant candidates and available grafts is exacerbated by varying organ allocation policies, which range from prioritizing urgency (“sickest-first”) to favoring candidates with better clinical conditions based on “individual transplant benefit” and “population-based transplant benefit” principles. Consideration of risk factors, such as high-risk donors and recipients, may lead to avoidance in clinical practice, impacting high-risk, waitlisted transplant candidates negatively (Calleja Lozano et al., 2022). Traditional scoring systems, like logistic regression, have limitations in organ transplantation, assuming linear relationships, overlooking key variables, and struggling with unbalanced problems. Modern biostatistics, relying on large cohorts, may lack accuracy in non-linear health sciences relationships. Incorporating AI enhances decision-making, ensuring optimal use and equal access despite organ scarcity challenges. AI-optimized allocation algorithms foster fair distribution, transcending geographical and socioeconomic barriers, contributing to more equitable and efficient organ transplantation systems (Briceño, 2020).

The use of AI in organ transplantation brings risks, including the accuracy-interpretability trade-off, methodological challenges, and a potential loss of explainability (Rana et al., 2008; Lisboa et al., 2022). Concerns extend to biases, accuracy, and acceptability of AI-driven decisions in clinical settings. Though accurate, AI models in organ transplantation often need more interpretability, making it challenging for clinicians and patients to understand. Methodological challenges in obtaining suitable transplant data for AI analysis need attention. Ensuring responsible AI integration in organ transplantation requires addressing these challenges. Collaboration and establishing criteria are essential to ensure responsible AI use in transplantation. Moreover, although promising, AI integration in organ transplantation introduces risks like algorithmic biases affecting organ allocation, compromising human-centric decision-making, and raising concerns about data privacy and security (Castillo-Astorga and Sotomayor, 2021). Ensuring transparency and accountability is crucial for the ethical implementation of AI in organ transplantation.

The potential risks of AI in organ utilization for transplantation encompass concerns about bias and accuracy, challenges in the clinical decision process, and criteria for AI acceptability. These factors currently hinder widespread AI adoption in transplantation. Extensive data integration poses risks of bias and inaccuracies, with AI potentially making predictions beyond existing literature. The lack of explainability in AI decision-making processes adds complexity, requiring efforts to enhance understanding. Establishing clinical and ethical acceptability criteria, forming a Transplant AI Team, and integrating AI into the Shared Decision-Making Model can address these challenges (Clement and Maldonado, 2021). Ensuring equity and avoiding biases in organ allocation is vital for fostering fairness and patient-centered care. Biases in allocation may lead to disparities, disadvantaging certain groups based on race or socio-economic status. Prioritizing equity helps guarantee equal access to life-saving organs, aligning with ethical principles of justice and beneficence. Fair allocation practices enhance public trust in the healthcare system and promote a more inclusive and ethical organ transplantation framework. By implementing transparent, unbiased protocols, healthcare providers can uphold the principle of justice, ensuring that organ allocation prioritizes medical needs rather than demographic factors.

### 4.1 AI in modern transportation

AI plays a pivotal role in modern transportation, with applications ranging from optimizing public transportation to enhancing traffic management. By leveraging AI algorithms, public transportation processes are automated, utilizing data from positioning devices, ticketing, and video surveillance to analyze network load and efficiency. AI contributes to traffic data collection, mitigating congestion, improving scheduling, and monitoring road conditions. AI extends to automated Mobility-as-a-Service (MaaS), optimizing individual transport through intelligent systems across vehicles, infrastructure, and management. These diverse applications showcase AI's capacity to address transportation challenges and enhance overall efficiency (Kamçi et al., 2019). The use of Internet of things (IoT) where a sensor attached to a donated kidney tracks its temperature and could ensure its preservation. This IoT-driven system optimizes the timely procurement of organs, providing accurate information like its exact location, estimated time of arrival to recipient hospital, kidney temperature, and its quality.

### 4.2 AI in transplant pathology

AI is poised to transform transplant pathology. Models like ChatGPT optimize pathologist's time for more meaningful tasks. AI tools enhance digital pathology, reducing errors and boosting efficiency. In transplant nephropathology, AI algorithms with whole-slide imaging (WSI) improve precision in identifying tissue components in pre-implantation kidney biopsies. Integrating AI enhances accuracy and workflow efficiency and eases pathologist workloads. AI models are trained to recognize rejection-related histopathological features and predict allograft survival by analyzing genetic factors like the APOL1 genotype (Rahman et al., 2023).

### 4.3 Future developments and transformative potential

Ongoing AI research in organ transplantation explores applications in organ allocation, donor-recipient pairing, and personalized immunosuppression, aiming to enhance overall transplant outcomes. The revolutionary potential of AI is evident in efforts to assess the quality of donated organs, potentially transforming the transplant system and saving lives. Furthermore, AI advancements in transplant pathology, using deep learning for various organs, indicate a growing focus on precision and efficiency in diagnostic processes. Integrating Smart Match and AI promises to reshape allocation dynamics, overcoming limitations for more precise matches and improved success rates. As AI transforms healthcare, Smart Match signifies progress toward efficiency, equitable distribution, and enhanced patient care, echoing AI expert Andrew Ng's view that “Artificial Intelligence is the new electricity”.

## Author contributions

RD: Conceptualization, Data curation, Formal analysis, Funding acquisition, Investigation, Methodology, Project administration, Resources, Software, Supervision, Validation, Visualization, Writing—original draft, Writing—review & editing.
